# Emotional availability: theory, research, and intervention

**DOI:** 10.3389/fpsyg.2015.01069

**Published:** 2015-07-28

**Authors:** Hannah Saunders, Allyson Kraus, Lavinia Barone, Zeynep Biringen

**Affiliations:** ^1^Department of Human Development and Family Studies, Colorado State University, Fort Collins, CO, USA; ^2^Department of Brain and Behavioral Science, Psychology Section, University of Pavia, Pavia, Italy

**Keywords:** emotional availability, attachment, intervention, emotional availability scales, psychology for clinical settings

## Abstract

Attachment theory (Bowlby, 1969) and its limitations are first described. Next, emotional availability (EA; Biringen et al., 1998; Biringen, 2008) is introduced as an expansion upon the original conceptualization of the parent–child attachment relationship. As a construct and as a measure, EA considers the dyadic and emotional qualities of adult–child relationships. EA is predictive of a variety of child outcomes, such as attachment security, emotion regulation, and school readiness. Recently developed programs to enhance adult–child EA are described.

## Attachment Theory and Research

Bowlby (1969) proposed attachment theory, which posits that the bond between a mother and her infant is based on an emotional connection. Attachment theory also argues that the attachment bond serves an evolutionary purpose, promoting the survival of the vulnerable infant by protecting him from danger and ensuring that his social and emotional needs are met (Bowlby, 1969). When an infant becomes fearful or distressed, his primary attachment figure serves as a source of comfort, and he learns to turn to that person in times of need. Furthermore, as the preference for the primary attachment figure develops, the infant also exhibits stranger anxiety, or fear and mistrust of unfamiliar adults. The emergence of such behaviors serves an evolutionary purpose because it parallels the infant’s increasing mobility, thus protecting the infant from potential dangers in the environment. Therefore, the infant uses his mother as a secure base as he explores and learns about his environment, “checking in” with her periodically.

## Attachment Styles

Ainsworth (1967) pioneered the first and most widely used measure of attachment, called the Strange Situation Procedure (SSP; Ainsworth et al., 1978), which assesses the attachment style of infants between the ages of 9 and 18 months. The procedure consists of several separation and reunion episodes with the mother, infant, and an adult stranger. The behaviors displayed by the infant during the separations and reunions are used to classify the infant into one of three styles: secure, insecure-avoidant, or insecure-anxious (Ainsworth et al., 1978). Infants with a secure attachment explore in the presence of their mother, protest when she leaves, regulate their emotions successfully during the separation, and greet the mother with joy when she returns. Infants with an insecure-avoidant attachment interact little with their mothers and react minimally when she leaves and returns. Infants with an insecure-anxious attachment explore the toys very little, are highly distressed when their mothers leave, and when mothers return, they approach her but angrily reject her comfort. Later research by Main and Solomon (1990) revealed a fourth attachment classification: disorganized. These infants behave unusually during the SSP, appearing disoriented, confused, detached, fearful, or angry. Disorganized attachment most often develops in cases of severe neglect, abuse, or domestic violence, but it is also seen in children with developmental disabilities and, less frequently, in normative samples (Cassidy and Shaver, 2008).

## Limitations of Attachment Theory and its Measures

Although attachment theory defines a parent–child bond as emotional, its assumptions largely focus on survival behaviors, and its most prominent assessment tool, the SSP, focuses entirely on infant behaviors during a mild stressful situation. However, the bond between a mother and her child certainly extends far beyond these behaviors. This fact is evident upon watching any mother–child pair interact. There are, of course, the predictable behaviors associated with survival-based attachment: infant exploration, periodic “check-ins” throughout the exploration, maternal comforting in distress, infant wariness of strangers, and infant distress upon separation. However, mothers and their infants also share an intense *emotional* connection. When the infant fusses, the mother furrows her brow in genuine concern and immediately looks for a way to remedy the distress; when the mother miraculously reappears from behind her hands in a game of peek-a-boo, the infant is gleefully surprised. These emotional expressions are not accounted for in the traditional attachment account because they extend beyond behaviors associated with separation. Furthermore, they do not only occur in stressful contexts like the separation-reunion paradigms of the Strange Situation. Rather, these emotions are seen in the regular, everyday, including positive and playful, interactions between mothers and their children. Therefore, the emotional connection in a caregiver-child relationship is clearly evident, and, furthermore, a healthy range of emotional expression is important to child development and well-being (Biringen and Easterbrooks, 2012).

Although attachment measures, such as the SSP, assess child behaviors and reactions to a caregiver, they rarely consider how child qualities evoke different behaviors in the caregiver. Each child is born with qualities that can alter how a mother (or father) responds to him. For example, studies suggest that infant irritability can evoke lower levels of sensitivity in the mother, contributing to a higher likelihood of insecure attachment (e.g., Susman-Stillman et al., 1996), and others conducted in the field of adoption show a significant and moderate effect of temperament on promoting children’s secure attachment (Lionetti, 2014). Thus, the development of healthy attachment bonds depends on the mother’s qualities as well as the child’s qualities, including temperament and other evocative effects.

The original conceptualization of attachment focused largely on the relationship between mothers and infants during the first year of life. In subsequent years, attachment theory has been expanded to attachment relationships in childhood (i.e., Waters et al., 1985; Main and Cassidy, 1988; Greenberg et al., 1993), in couples with atypical parental roles such as adoptive parents (Lionetti et al., 2015), in adulthood (i.e., George et al., 1984), and between romantic partners (Tatkin, 2005; Johnson, 2012). However, the assessments used for infants, children, and adults differ significantly in their methods. Furthermore, although attachment theory assumes that an individual’s attachment style remains stable across the lifepan, a recent longitudinal study (Groh et al., 2014) found no evidence of continuity from infant attachment classification to adult attachment classification. This lack of continuity could be due to lawful discontinuity (Weinfield et al., 2000), or because of changes in measures. Therefore, no single attachment measure can account for the quality of relationships across the lifespan.

Finally, attachment theory focuses largely on the parent–child relationship, so it does not account for the quality of other relationships in children’s lives. For example, children frequently interact with teachers, siblings, babysitters, and friends, and these relationships have an increasing effect on children’s lives as they get older. However, viewing all of these significant relationships as attachment *per se* may not be accurate.

Additionally, many family theories emphasize the importance of viewing the family as a dynamic system, with each member affecting the other and the larger system (e.g., Bateson, 1972; Haley, 1976; Whitaker and Bumberry, 1988; Guttman, 1991; Satir et al., 1991). While attachments can and should be viewed from a family systems lens, it becomes difficult to understand the contributions of each family member, when the measures represent the behaviors and/or views of one individual member (e.g., SSP). Thus, attachment theory’s focus on the individual’s behaviors and/or views limits its potential to assess the wide variety of relationships in children’s lives, as well as the complexity of the family as a whole.

## Emotional Availability

Emotional availability (EA) refers to the ability of two people to share a healthy emotional connection, and it thus elucidates the emotional and dyadic quality of relationships. It expands upon the behaviors associated with attachment by including the dyadic, emotional, and structural characteristics of a relationship. The dyadic quality of EA considers the perspectives of both the adult and child, rather than prescribing specific behaviors that may be influenced by cultural biases. This characteristic allows it to be observed and measured in any context or culture. Additionally, because EA considers the emotional climate of the relationship, it offers richer information about the relationship. The EA framework also accounts for the adult’s ability to provide structure within the relationship by guiding the child’s learning and supporting his or her autonomy. Furthermore, EA can be observed across a wide range of child ages, from birth to age 14 (Biringen and Easterbrooks, 2012). Theoretically, the system can also be used beyond this age period.

Although the term “emotional availability” has been used in the field of psychological research since the 1970s (Mahler et al., 1975), a validated measure of the construct was only developed in the last 20 years. The EA assessment, developed by Biringen et al. (1998) and Biringen (2008), consists of six scales, four of which measure the adult’s emotions and behaviors, and two of which measure the child side of the interaction. The adult dimensions are sensitivity, structuring, non-intrusiveness, and non-hostility. The child dimensions are responsiveness and involvement. Each dimension is measured using a Likert-type continuous scale that assigns a score between 1 and 7. Assessing the perspective of both the adult and the child is beneficial, both to reflect that adult–child relationships are bidirectional, as well as to capture any possible differences between the adult and child.

Sensitivity consists of the behaviors and emotions used by an adult to create and maintain a positive, healthy emotional connection with the child. Recent research in neuroscience indicates that infants of sensitive mothers (using the EA system) are more responsive to happy than neutral faces (Taylor-Colls and Fearon, 2015). This finding is consistent with the emphasis of the EA system not only on response to stress but also to enjoyable times.

Structuring refers to the capacity of an adult to support the child’s learning and guide him or her toward a higher level of understanding. An optimally structuring adult not only teaches and helps the child, but also permits a degree of autonomy so that the child can learn independently. In order to be successful, the adult must meet the child at his or her current level of understanding and use both verbal and non-verbal strategies to guide the child.

Non-intrusiveness refers to the ability of an adult to follow the child’s lead during play and avoid interfering. A non-intrusive adult does not interrupt the child physically or verbally, limits commands, permits the child age-appropriate levels of independence, and withdraws when the child is seeking such independence.

Non-hostility refers to whether or not the adult is able to regulate his or her own negative emotions to avoid expressing these toward the child. A failure to effectively regulate emotions leads to the adult demonstrating covert and/or overt hostility. Covert hostility consists of the less-obvious expression of negative emotions, such as impatience, frustration, and boredom. Overt hostility consists of behaviors such as negative statements toward the child, physical aggression, and threats of separation.

Child responsiveness to the adult and child involvement of the adult encompass the child’s degree of EA with the adult. A highly responsive child interacts with the adult when she reaches out and clearly enjoys doing so. A highly involving child invites the adult to join her play and talks to the adult. Both responsiveness and involvement are balanced with the child’s desire to pursue autonomy and explore the environment. Furthermore, children who are appropriately involving and responsive rarely connect with the adult through negative emotions and behaviors, such as anxiety, whining, throwing tantrums, or acting out. Thus, the child’s side of the relationship is an important clue to overall relationship health, one that is not often available by only observing the parent’s side of the relationship.

The six dimensions of EA account for the dyadic quality of parent–child relationships and the variety of behaviors and emotions of this quality. Thus, we argue that adult sensitivity is not the only factor that contributes to the relationship’s health. Interestingly, a recent study by Licata et al. (2015) found that child involvement was related to maternal sensitivity and higher left frontal activation of the brain, as measured with the electroencephalogram. However, child responsiveness was related to maternal sensitivity, but not neurological activation. Thus, this study shows the importance of differentiating among EA dimensions, as well as how the complexity of parent–child interactions extends beyond attachment behaviors.

Emotional availability is a broad-based, easily applicable, and user-friendly way to understand a myriad of relationships (Biringen et al., 2014). While all six dimensions of EA are important in the description of the overall quality of the parent–child relationship, the system also summarizes these six qualities and offers a measure of attachment. This measure of attachment is the Emotional Attachment and EA Clinical Screener (EA2-CS). EA2-CS is scored on a 100-point scale, divided into 4 categorical zones (Emotionally Available; Complicated; Detached; and Problematic/Disturbed) that map onto the four attachment categories. Early studies on the EA2-CS show that it is associated with attachment styles, as measured by the Attachment Q-Sort (Baker and Biringen, 2012) and the Diagnostic Classification 0-3 Parent-Infant Relationship Global Assessment Scale (DC 0-3 PIRGAS; Espinet et al., 2013). Recent studies have been testing—through randomized control trials with attachment-based interventions—its contribution in assessing positive parenting in adoptive families (Barone et al., 2015). A paper on the relations between the EA2-CS relations and the Adult Attachment Interview and the SSP is in progress.

## Can the Parent Look Good Without the Child?

In a dyadic relationship, the participants influence each other in a bidirectional manner. Sometimes parents are very sensitive and responsive, but the child may not react accordingly. Biringen et al. (1998) argued that, essentially, the parent cannot be considered highly sensitive unless the child is emotionally responsive to him or her. However, parental qualities as well as child qualities are certainly viewed in their own right. A child who avoids a well-meaning, positive mother can be given low scores, while such a mother would show a much higher profile of scores. In two studies on adoptive families, often the child and parent EA scores were quite different (Baker et al., 2015; Barone et al., 2015). In fact, Barone et al. (2015) reported that in 22% of the adoptive dyads each member scored in a different EA2-CS zone from the adoptive mother.

## Emotional Availability and Child Outcomes

Emotional availability in parent–child relationships predicts a wide range of child outcomes. First, EA significantly relates to child attachment security, both with parents and professional caregivers (Easterbrooks and Biringen, 2000; Altenhofen et al., 2013).

Additionally, EA has been linked to child emotion regulation. Specifically, in a sample of low-income mother–child pairs, children who experienced higher EA in their relationship demonstrated superior emotional control in a challenging situation (Little and Carter, 2005). Another study found that higher levels of sensitivity predict better regulation of stress responses among highly inhibited children (Kertes et al., 2009).

A longitudinal study of EA (Moreno et al., 2008) found that maternal EA at 15 months predicted child expressive language abilities and child EA at age two. Additionally, child EA at age two predicted child empathy toward both the mother and other adults at age four (Moreno et al., 2008). Studies on EA in preschool-aged children have demonstrated that higher parent–child EA predicts fewer problems and higher social competence in preschool and during the transition to kindergarten (Biringen et al., 2005; Howes and Hong, 2008). Specifically, in a study by Biringen et al. (2005) higher mother–child EA in the year leading up to kindergarten predicted lower child aggression, victimization, internalizing problems, and externalizing problems during the transition to kindergarten. Furthermore, in a sample of Mexican-heritage families in the U.S., mothers’ sensitivity and structuring when the child was three predicted children’s pretend play and social competence during preschool (Howes and Hong, 2008). These studies, among many others, have demonstrated that EA is predictive of a variety of child developmental outcomes.

## Emotional Availability in Other Relationships

Emotional availability lends itself well to research on a variety of different relationships. First, the construct can easily be applied to relationships in families. Family systems theory (Bateson, 1972; Haley, 1976) views families as dynamic systems in which individuals interact to influence one another and the family as a whole. EA accounts for these dynamic interactions between members in the context of the family system (e.g., mother with child 1, mother with child 2, father with child 1, father with child 2, even mother with father, and so on, Biringen, 2008), albeit at the dyadic levels within the larger family system.

Emotional availability encompasses more than parent–child relationships. For example, a group in Sweden is investigating the therapist-client relationship in terms of EA (Söderberg et al., 2013). Other studies are examining EA in romantic couples (e.g., Derr-Moore, 2015). Recent therapist and couples conceptualizations and versions facilitate this work (cf. Biringen, 2008).

## Interventions

Numerous studies using a variety of prevention/intervention approaches have investigated whether EA can be altered; see Biringen et al. (2014) for a systematic review. Most recently, a longitudinal randomized control trial study testing the effectiveness of the Video Feedback Intervention for promoting Positive Parenting and Sensitive Discipline (VIPP-SD; Juffer et al., 2008) in adoptive families found a significant effect of the VIPP-SD on mother–child EA in the first 2 years after adoption (Barone et al., 2015). In a separate study with adoptive families, Baker et al. (2015) used the EA Intervention with group-format distance technology (i.e., Skype) to connect the mothers to the facilitator as well as group members. The study documented enhancement in maternal perceptions as well as observed EA between adoptive mother and child. Both studies demonstrate a growing awareness and promise of evidence-based post-adoption programming and the feasibility of altering EA in relationships where a child’s signals may be difficult to interpret.

Additional implementation of the EA Intervention has been reported with low SES and high SES groups using an in-person group format with findings of lower parental stress and/or depressive symptoms, as well as enhanced observed EA (Biringen et al., 2010). The program was also implemented in-person with childcare professionals in a one-on-one coaching context; in this study, the treatment group showed significant improvements in adult–child EA and attachment style as compared to a non-treatment control (Biringen et al., 2012).

An additional program to enhance EA in the family system is called *Love Now, Success Later (LNSL)* and is currently being tested. This program targets couples when mothers are in their third trimester of pregnancy. This program includes a video-based educational component about attachment and EA, as well as mindfulness practice, such as 3-min breathing and kindness practice. The mindfulness practices help individuals regulate negative affectivity and stress during pregnancy and the postpartum period. The goal of LNSL is to build expectant mothers’ and fathers’ skills that will help them promote a secure attachment and high levels of EA with one another and with the new baby; those who have participated report high levels of engagement and satisfaction. We are interested in whether the program will help regulate the stresses of pregnancy and enhance attachment to the unborn baby as well as prepare couples as a family unit for the challenges of the postpartum period. We are also interested in whether the emphasis on stress regulation through mindfulness practice may lead to babies who are easier to be with (in the sense of crying, feeding, and sleeping).

## Conclusion

The field of attachment research acknowledges that there are many important aspects of parent–child relationships. The various dimensions of EA can serve to capture these additional aspects. Including EA as an indicator of the quality of parent–child relationship allows for the behavior of both the parent and child to be measured, with acknowledgment that the view of the parent may not be the view of the child on all occasions. Including this construct in a battery of assessments provides both a measure of parent–child relationship quality as well as a new measure of the attachment. This framework also has been useful in intervention work to promote parent and child well being.

### Conflict of Interest Statement

The authors declare that the research was conducted in the absence of any commercial or financial relationships that could be construed as a potential conflict of interest.

## References

[B1] Ainsworth M. (1967). Infancy in Uganda. Baltimore: Johns Hopkins.

[B2] Ainsworth M. D. S. Blehar M. Waters E. Wall S. (1978). Patterns of Attachment: A Psychological Study of the Strange Situation. Hillsdale, NJ: Erlbaum.

[B3] Altenhofen S. Clyman R. Little C. Baker M. Biringen Z. (2013). Attachment security in three-year-olds who entered substitute care in infancy. Infant Ment. Health J. 34, 435–445. 10.1002/imhj.21401 PMC4846274 27127309

[B4] Baker M. Biringen Z. (2012). Emotional Attachment and Emotional Availability Clinical Screener. Los Angeles, CA: National Training Institute.

[B5] Baker M. Biringen Z. Schneider A. Meyer-Parsons B. (2015). An emotional-availability-based tele-intervention for adoptive families. Infant Ment. Health J. 36, 179–192. 25704237 10.1002/imhj.21498

[B6] Bateson G. (1972). Steps to An Ecology of Mind. San Francisco, CA: Chandler.

[B7] Barone L. Lionetti F. Dellagiulia A. Alagna C. Rigobello L. (2015). “Promoting emotional availability in mothers of late adopted children: a randomized controlled trial using the VIPP-SD,” in Paper Presented at the 7th International Attachment Conference, New York, August 6–8.

[B8] Biringen Z. (2008). The Emotional Availability (EA) Scales Manual, 4th Edn. Boulder, CO: International Center for Excellence in Emotional Availability.

[B9] Biringen Z. Altenhofen S. Aberle J. Baker M. Brosal A. Bennett S. (2012). Emotional availability, attachment, and intervention in center-based child care for infants and toddlers. Dev. Psychopathol. 24, 23–34. 10.1017/S0954579411000630 22292991

[B10] Biringen Z. Batten R. Neelan P. Altenhofen S. Swaim R. Bruce A. (2010). Emotional availability (EA): the assessment of and intervention for global parent–child relational quality. J. Exp. Psychother. 49, 3–9.

[B11] Biringen Z. Derscheid D. Vliegen N. Closson L. Easterbrooks A. E. (2014). Emotional availability (EA): theoretical background, empirical research using the EA Scales, and clinical applications. Dev. Rev. 34, 114–167. 10.1016/j.dr.2014.01.002

[B12] Biringen Z. Easterbrooks M. A. (2012). The integration of emotional availability into a developmental psychopathology framework: reflections on the special section and future directions. Dev. Psychopathol. 24, 137–142. 10.1017/S0954579411000733 22396974

[B13] Biringen Z. Robinson J. Emde R. (1998). Emotional Availability Scales, 3rd Edn. Unpublished Manual for the EAS-training. Available at: www.emotionalavailability.com 10.1080/14616730050085617 11707915

[B14] Biringen Z. Skillern S. Mone J. Pianta R. (2005). Emotional availability is predictive of the emotional aspects of childrens’ “school readiness”. J. Early Child. Infant Psychol. 1, 81–97.

[B15] Bowlby J. (1969). Attachment and Loss, Vol. 1. London: Basic Books.

[B16] Cassidy J. Shaver P. R. (2008). Handbook of Attachment: Theory, Research, and Clinical Applications. New York: Guilford Press.

[B17] Derr-Moore A. (2015). Mindfully and Emotionally Intervening on the Rocky Road to Parenthood: A Parental and Couples Perspective. Unpublished master’s thesis, Colorado State University, Fort Collins.

[B18] Easterbrooks M. A. Biringen Z. (2000). Mapping the terrain of emotional availability and attachment. Attach. Hum. Dev. 2, 129–135. 10.1080/14616730050085518 11707906

[B19] Espinet S. D. Jeong J. J. Motz M. Racine N. Major D. Pepler D. (2013). Multimodal assessment of the mother–child relationship in a substance-exposed sample: divergent associations with the emotional availability scales. Infant Ment. Health J. 34, 496–507.

[B20] Haley J. (1976). Problem-Solving Therapy: New Strategies for Effective Family Therapy. San Francisco, CA: Jossey-Bass.

[B21] Howes C. Hong S. S. (2008). Early emotional availability: predictive of pre-kindergarten relationships among Mexican heritage children? J. Early Child. Infant Psychol. 4, 4–26.

[B22] George C. Kaplan N. Main M. (1984). The Adult Attachment Interview. Berkeley, CA: University of California.

[B23] Greenberg M. T. Cicchetti D. Cummings E. M. (1993). Attachment in the Preschool Years: Theory, Research, and Intervention. Chicago: University of Chicago Press.

[B24] Groh A. M. Roisman G. I. Booth-LaForce C. Fraley R. C. Owen M. T. Cox M. J. (2014). Stability of attachment security from infancy to late adolescence. Monogr. Soc. Res. Child Dev. 79, 51–66. 10.1111/mono.12113 25100089 PMC13245647

[B25] Guttman H. A. (1991). “Systems theory, cybernetics and epistemology,” in Handbook of Family Therapy, Vol. 2, eds Gurman A. S. Kniskern D. P. (New York, NY: Brunner/Mazel), 41–64.

[B26] Johnson S. M. (2012). Practice of Emotionally Focused Couple Therapy: Creating Connection. New York, NY: Routledge.

[B27] Juffer F. Bakermans-Kranenburg M. J. van IJzendoorn M. H. (2008). Promoting Positive Parenting: An Attachment-Based Intervention. New York: Taylor & Francis.

[B28] Kertes D. A. Donzella B. Talge N. M. Garvin M. C. van Ryzin M. J. Gunnar M. G. (2009). Inhibited temperament and parent emotional availability differentially predict young children’s cortisol responses to novel social and non-social events. Dev. Psychobiol. 51, 521–532. 10.1002/dev.20390 19676107 PMC5870881

[B29] Licata M. Paulus M. Kuhn-Popp N. Meinhardt J. Sodian B. (2015). Infant frontal asymmetry predicts child emotional availability. Int. J. Behav. Dev. 10, 1–5. 10.1177/0165025415576816

[B30] Lionetti F. (2014). What promotes secure attachment in early adoption? The protective roles of infants’ temperament and adoptive parents’ attachment. Attach. Hum. Dev. 16, 573–589. 10.1080/14616734.2014.959028 25255300

[B31] Lionetti F. Pastore M. Barone L. (2015). Parenting stress: the roles of attachment states of mind and parenting alliance in the context of adoption. Parenting 15, 75–91. 10.1080/15295192.2015.1020142

[B32] Little C. Carter A. (2005). Negative emotional reactivity and regulation in 12-month-olds following emotional challenge: contributions of maternal-infant emotional availability in a low-income sample. Infant Ment. Health J. 26, 354–368. 10.1002/imhj.20055 28682470

[B33] Mahler M. Pine F. Bergman A. (1975). The Psychological Birth of the Human Infant. New York: Basic.

[B34] Main M. Cassidy J. (1988). Categories of response to reunion with the parent at age 6: predictable from infant attachment classifications and stable over a 1-month period. Dev. Psychol. 24, 415–426. 10.1037/0012-1649.24.3.415

[B35] Main M. Solomon J. (1990). “Procedures for identifying infants as disorganized/disoriented during the Ainsworth Strange Situation,” in Attachment in the Preschool Years: Theory, Research, and Intervention, Vol. 1, eds Greenberg M. T. Cicchetti D. Cummings E. M. (Chicago, University of Chicago Press), 121–160.

[B36] Moreno A. J. Klute M. M. Robinson J. L. (2008). Relational and individual resources as predictors of empathy in early childhood. Soc. Dev. 17, 613–637. 10.1111/j.1467-9507.2007.00441.x

[B37] Satir V. Banmen J. Gerber J. Gomori M. (1991). The Satir Model: Family Therapy and Beyond. Palo Alto, CA: Science and Behavior Books.

[B38] Söderberg A. K. Elfors C. Holmqvist Larsson M. Falkenström F. Holmqvist R. (2013). Emotional availability in psychotherapy: the usefulness and validity of the Emotional Availability Scales for analyzing the psychotherapeutic relationship. Psychother. Res. 24, 91–102. 10.1080/10503307.2013.826833 23957271

[B39] Susman-Stillman A. Kalkoske M. Egeland B. Waldman I. (1996). Infant temperament and maternal sensitivity as predictors of attachment security. Infant Behav. Dev. 19, 33–47. 10.1016/S0163-6383(96)90042-9

[B40] Tatkin S. (2005). Marital therapy and the psychobiology of turning toward and turning away. Therapist 15, 75–78.

[B41] Taylor-Colls S. Fearon R. M. P. (2015). The effects of parental behavior on infants’ neural processing of emotion expressions. Child Dev. 86, 877–888. 10.1111/cdev.12348 25676831

[B42] Waters E. Deane K. Bretherton I. Waters E. (1985). Q-methodology and the organization of behavior in infancy and early childhood. Growing pains of attachment theory and research. Monogr. Soc. Res. Child Dev. 50, 41–65.

[B43] Weinfield N. S. Sroufe L. A. Egeland B. (2000). Attachment from infancy to young adulthood in a high-risk sample: continuity, discontinuity and their correlates. Child Dev. 71, 695–702. 10.1111/1467-8624.00178 10953936

[B44] Whitaker C. A. Bumberry W. M. (1988). Dancing With the Family. New York: Brunner/Mazel.

